# Correction: Hafnium–zirconium oxide interface models with a semiconductor and metal for ferroelectric devices

**DOI:** 10.1039/d1na90069b

**Published:** 2021-08-09

**Authors:** Kisung Chae, Andrew C. Kummel, Kyeongjae Cho

**Affiliations:** Department of Chemistry and Biochemistry, University of California San Diego La Jolla CA USA akummel@ucsd.edu; Department of Materials Science and Engineering, The University of Texas at Dallas Richardson TX USA kjcho@utdallas.edu

## Abstract

Correction for ‘Hafnium–zirconium oxide interface models with a semiconductor and metal for ferroelectric devices’ by Kisung Chae *et al.*, *Nanoscale Adv.*, 2021, DOI: 10.1039/d1na00230a.

The authors regret that an incorrect version of Fig. 7 was included in the original article. The correct version of Fig. 7 is presented below.

**Fig. 7:**
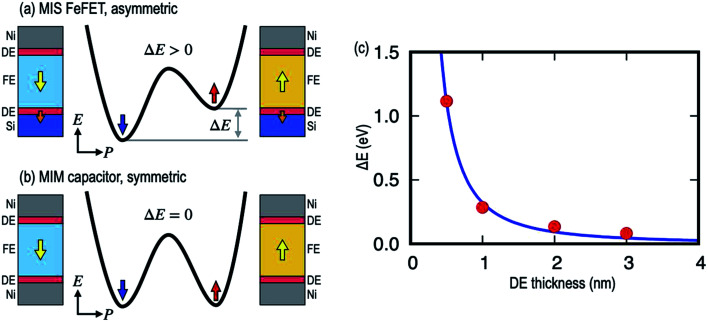
Total energy landscape in MIM and MIS devices. Schematic diagram of energy landscapes as a function of polarization state for (a) an asymmetric MIS FeFET and (b) a symmetric MIM capacitor. (c) The energy difference (Δ*E*) as a function of DE thickness in MIS with the FE thickness fixed at 2 nm.

The Royal Society of Chemistry apologises for these errors and any consequent inconvenience to authors and readers.

## Supplementary Material

